# Supplementary material for: Assembly and performance of a cholera RDT prototype that detects both Vibrio cholerae and associated bacteriophage as a proxy for pathogen detection
Source: J Clin Microbiol. 2024 Dec 31;63(2):e01443-24. doi: 10.1128/jcm.01443-24 (PMC11837499; doi:10.1128/jcm.01443-24)
Supplement: Supplemental material — Figures S1 to S8; Tables S1 to S3. [file jcm.01443-24-s0002.docx]

**SUPPLEMENTARY MATERIALS**


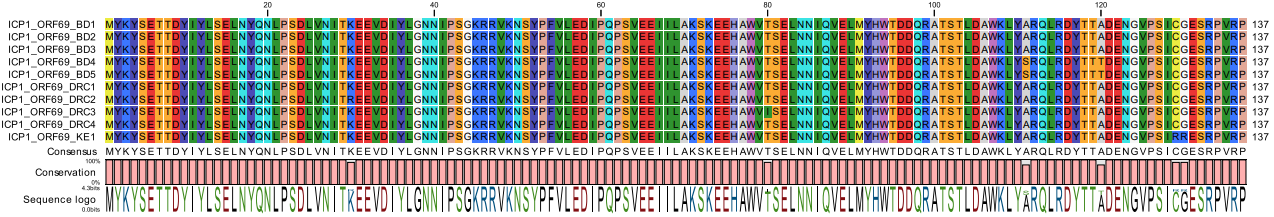


**B.**


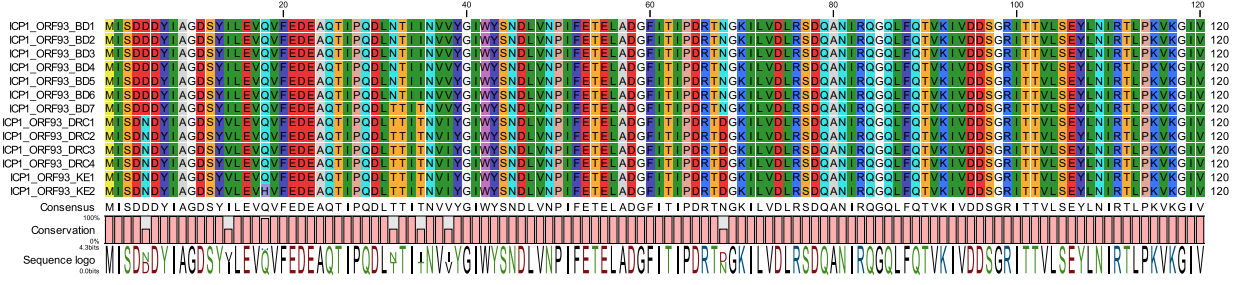


**C.**


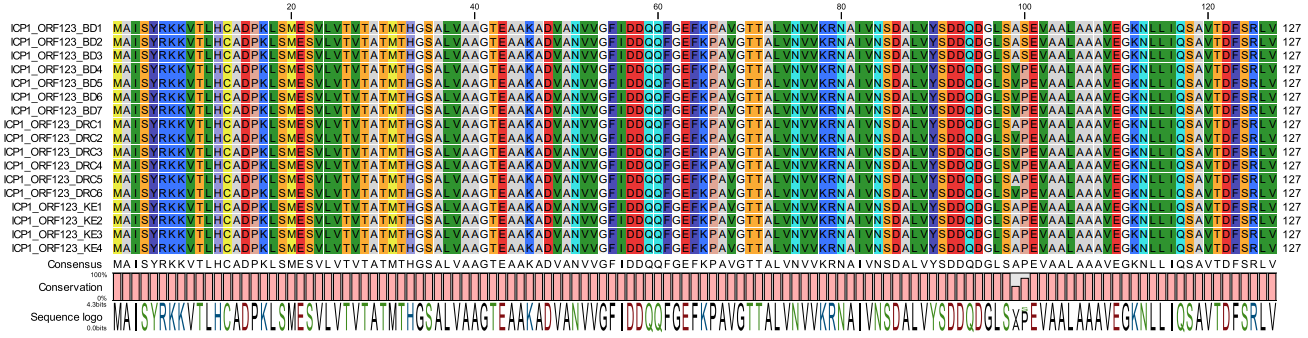


**Figure S1** Amino acid alignment of ICP1 tail fiber ORF69 (**A**), tail fiber ORF93 (**B**), and capsid decoration protein ORF123 (**C**) from Bangladesh (BD), Democratic Republic of Congo (DRC), and Kenya (KE). Sequences were obtained by PCR amplification and sequencing of clinical samples. Data visualizations prepared in QIAGEN CLC.


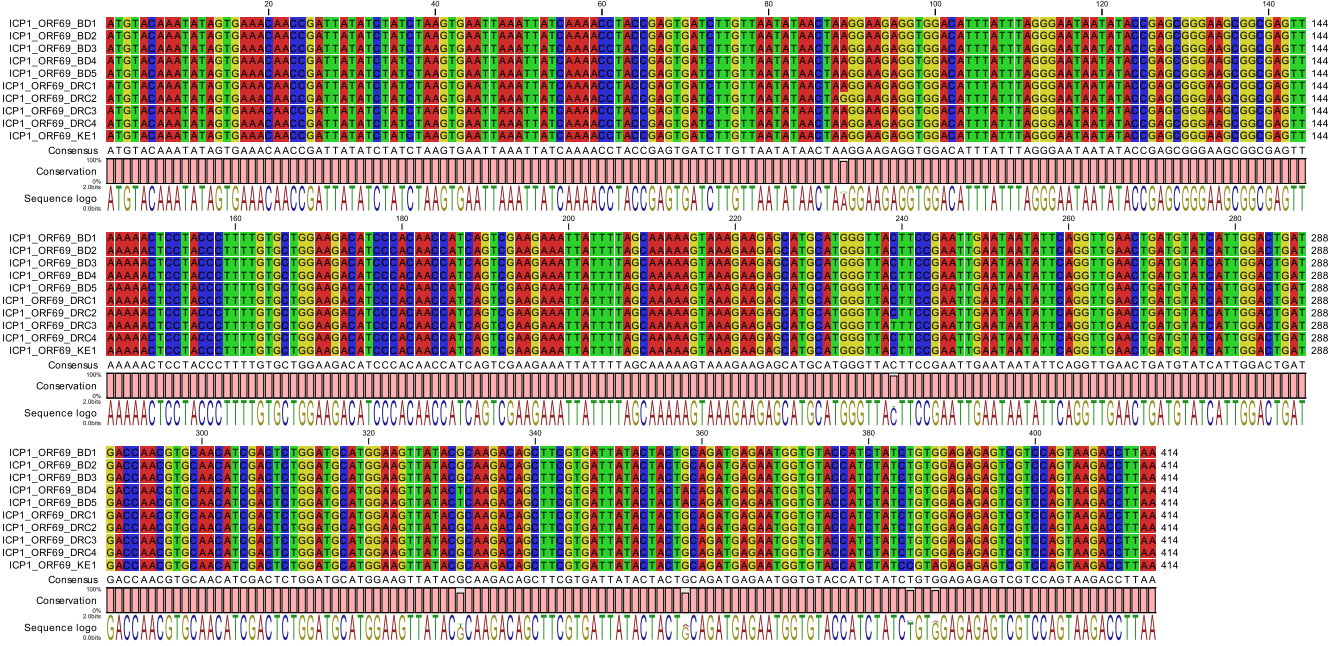


**Figure S2** Nucleic acid alignment of ICP1 tail fiber ORF69 from Bangladesh (BD), Democratic Republic of Congo (DRC), and Kenya (KE). Sequences were obtained by PCR amplification and sequencing of clinical samples. Data visualizations prepared in QIAGEN CLC.


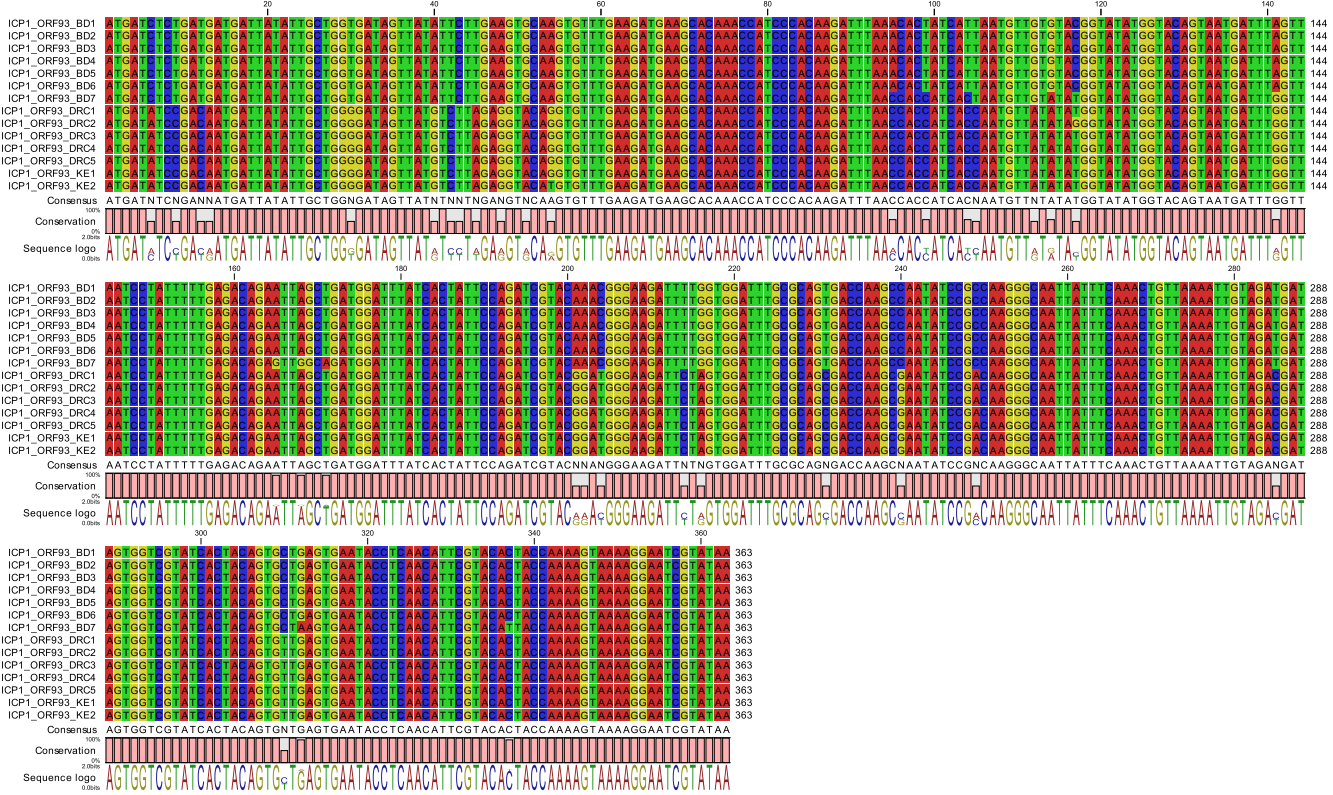


**Figure S3** Nucleic acid alignment of ICP1 tail fiber ORF93 from Bangladesh (BD), Democratic Republic of Congo (DRC), and Kenya (KE). Sequences were obtained by PCR amplification and sequencing of clinical samples. Data visualizations prepared in QIAGEN CLC.


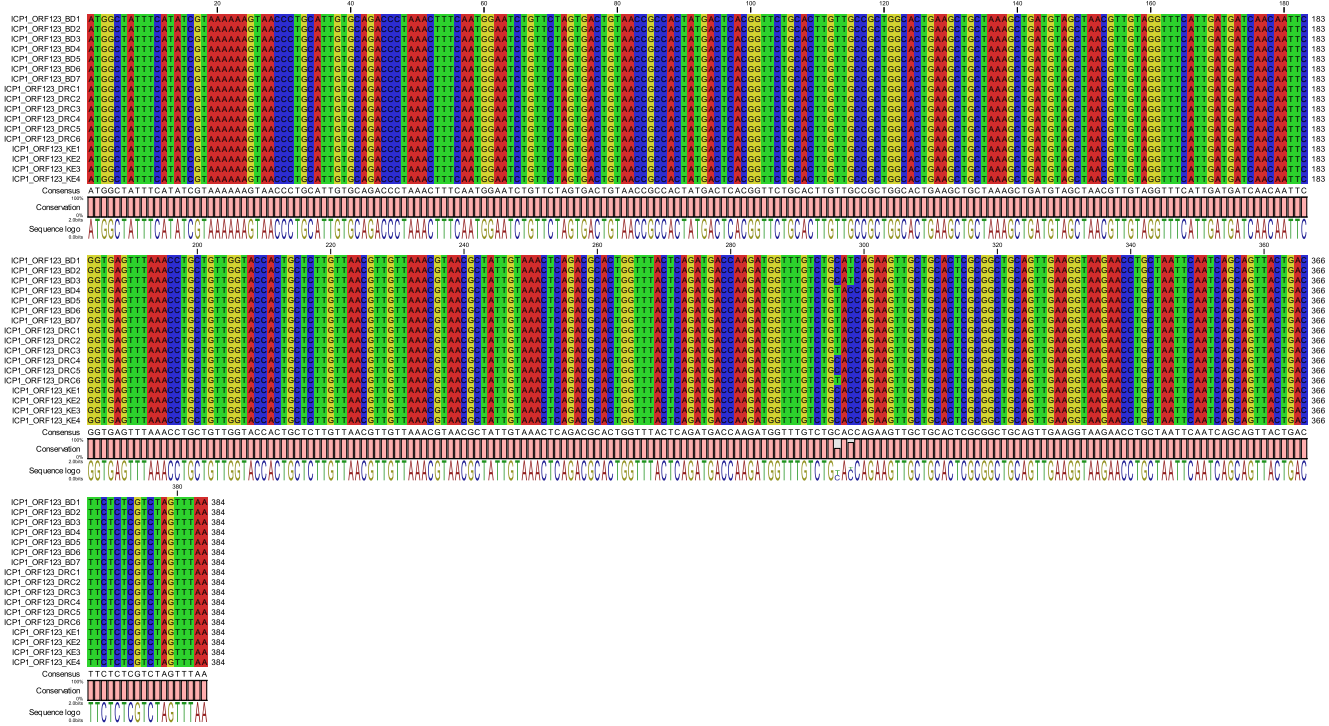


**Figure S4** Nucleic acid alignment of ICP1 head decoration protein from Bangladesh (BD), Democratic Republic of Congo (DRC), and Kenya (KE). Sequences were obtained by PCR amplification and sequencing of clinical samples. Data visualizations prepared in QIAGEN CLC.


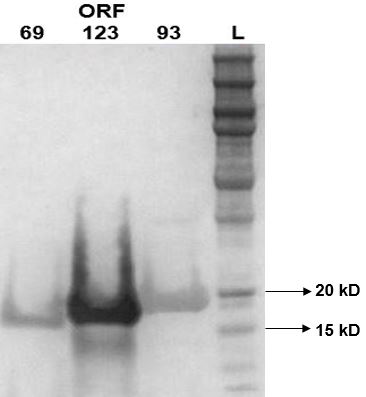


**Figure S5** Western blot of His-tagged tail fiber ORF69, head decoration protein ORF123 and tail fiber ORF93 after purification. L = protein marker ladder.


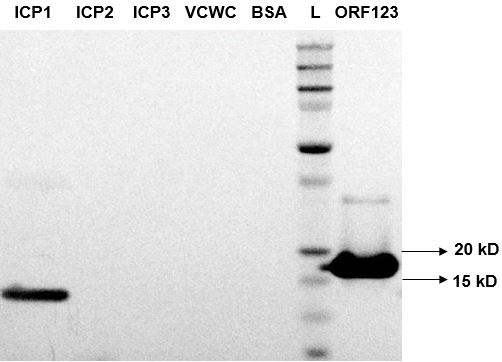


**Figure S6** Western blot analysis of candidate ICP1 head decoration protein ORF123_mAbCL14 against ICP1. Similar results were observed for ICP1ORF123_mAbCL15 and CL16 (not shown here). Negative controls are ICP2 and ICP3. VCWC = formalin-killed *V. cholerae* whole-cell, bovine serum albumin = BSA. L = ladder (protein marker), ORF123 = ICP1 recombinant proteins (positive control).


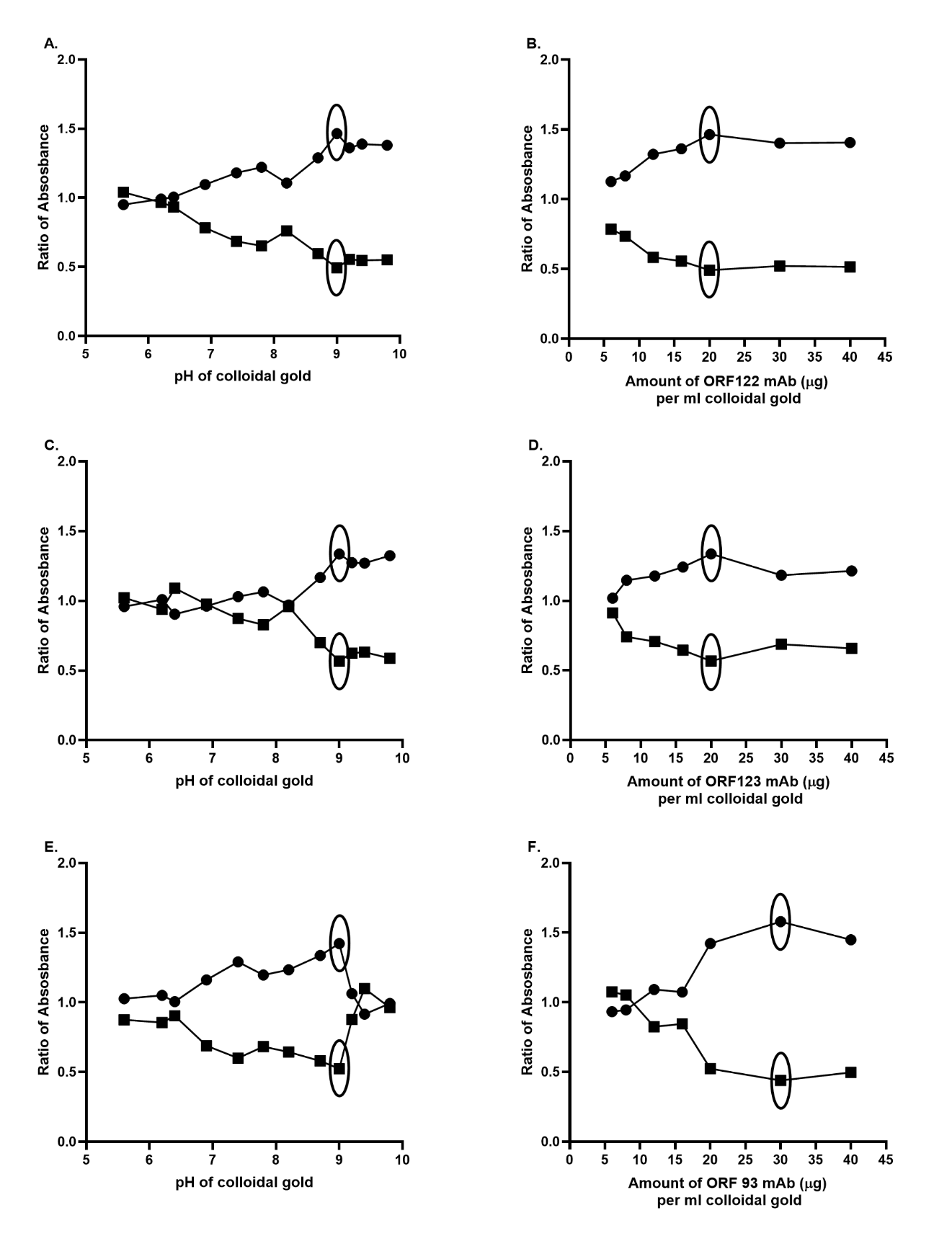


**Figure S7** Optimization of pH and minimum mAb concentration for ORF122 mAb (A,B), ORF123 mAb (C,D) and ORF93 mAb (E,F) gold conjugation by aggregation test. Here, a ratio of absorbance at 520 nm and 580 nm represents stability, and the ratio of 600 nm to 520 indicates polydispersity of conjugated gold solution.


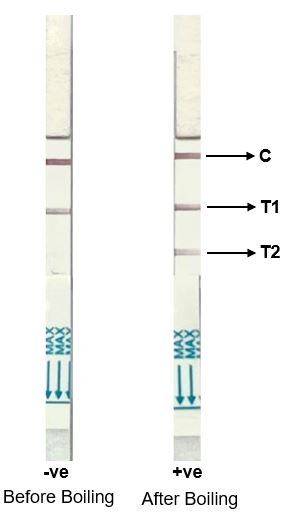


**Figure S8** Test results of ICP1 RDTplus for the detection of both cholera LPS and ICP1. RDTplus was tested with 1:10 diluted native and boiled ICP1. Here, C = control line, T1= test line for VC LPS, T2=test line for ICP1, -ve= negative result and +ve=positive result. Red lines indicate positive control or test line. Positive C line ensures the validity of the RDT prototype result.

| **Targets** | **Primer sequences** |
| --- | --- |
| ORF 69 (tail fiber protein) | Forward: 5’-ATGTACAAATATAGTGAAACAACCG-3’ |
|  | Reverse: 5’-TTAAGGTCTTACTGGACGACTC-3’ |
| ORF 93 (tail fiber protein) | Forward: 5’-ATGATCTCTGATGATG-3’ |
|  | Reverse: 5’-TTATACGATTCCTTTTACTTTTGGTAG-3’ |
| ORF 123 (head decoration protein) | Forward: 5’-ATGGCTATTTCATATCG-3’ |
|  | Reverse: 5’-TTAAACTAGACGAGAGAAGTC-3’ |

**Table S1** List of primers used in molecular analysis.

**Table S2** Comparison of new target amino acid and nucleic acid sequences from Bangladesh and DRC ICP1 isolate genomic sequences (1, 2).

| **Targets** | **Length (bp)** | **Mass (Da)** | **Nucleic Acid similarity (%)** | **Amino Acid similarity (%)** |
| --- | --- | --- | --- | --- |
| ORF 69  (tail fiber protein) | 414 | 15,966 | 99.5 | 98.5 |
| ORF 70  (tail fiber protein) | 1068 | 38,096 | 98.9 | 97.0 |
| ORF 84  (tail sheath protein) | 1392 | 50,897 | 91.7 | 95.0 |
| ORF 93  (tail fiber protein) | 363 | 13,537 | 92.6 | 96.0 |
| ORF 123 (head decoration protein) | 384 | 13,193 | 99.7 | 99.2 |

**Table S3** Comparison of new target amino acid and nucleic acid sequences obtained by PCR and sequencing of ICP1 positive stool samples in Bangladesh, DRC and Kenya.

| **Targets** | **Length (bp)** | **Mass (Da)** | **Nucleic Acid similarity (%)** | **Amino Acid similarity (%)** |
| --- | --- | --- | --- | --- |
| ORF 69  (tail fiber protein) | 414 | 15,966 | 99.5-100 | 98.5-100 |
| ORF 93  (tail fiber protein) | 363 | 13,537 | 90.4-100 | 94-100 |
| ORF 123  (head decoration protein) | 384 | 13,193 | 99.4-100 | 98.4-100 |

**REFERENCES**

1. **Alam MT, Mavian C, Paisie TK, Tagliamonte MS, Cash MN, Angermeyer A, Seed KD, Camilli A, Maisha FM, Senga RKK, Salemi M, Morris JG, Jr., Ali A.** 2022. Emergence and Evolutionary Response of *Vibrio cholerae* to Novel Bacteriophage, Democratic Republic of the Congo(1). Emerg Infect Dis **28:**2482-2490.

2. **LeGault KN, Hays SG, Angermeyer A, McKitterick AC, Johura FT, Sultana M, Ahmed T, Alam M, Seed KD.** 2021. Temporal shifts in antibiotic resistance elements govern phage-pathogen conflicts. Science **373**.
